# Round the Hospitals

**Published:** 1920-04-03

**Authors:** 


### ROUND THE HOSPITALS.

The King took occasion to visit the Grangethorpe Ministry of Pensions Hospital during his visit to Manchester on March 27, and having inspected the building where orthopædic and other cases are under treatment, he talked with much sympathy and kindness to the patients. Guards of honour were formed by the wounded and convalescent and by the nurses. At the end of his visit the King conferred the Royal Red Cross upon Sister Moffatt and three other nursing sisters. His Majesty also visited the Labour Ministry's training-centre at the Manchester City Hall, where wonderful things are being done to fit disabled Service men for a life of independence in the teeth of terrible physical drawbacks. The King was much interested in Mr. John Taylor, late of the 2nd Seaforths, who, in spite of the loss of both legs, moves about with ease in a mechanical chair, and has become an excellent watch and clock worker.

**ROUND THE HOSPITALS**—(*continued*).

The King held two Investitures at Buckingham Palace last week. On March 24 he conferred decorations on the following members of the nursing services:—Bar to the Royal Red Cross: Miss Clara Chadwick, Miss Lavinia Steen, and Miss Sarah Wilshaw, Q.A.I.M.N.S.; Royal Red Cross, 1st Class: Misses M. Clements and A. Plimsaul, Q.A.I.M.N.S.; Mrs. Charlotte Craven, Miss E. Gascoigne, and H. Walde, Q.A.I.M.N.S. (R.); Miss M. Cummins; Miss J. Hannah, T.F.N.S.; Miss G. Montgomery, C.N.S.; Miss M. Whent, B.R.C.S.; Miss A. Duncan, Civil Hospital Reserve. Royal Red Cross, 2nd Class: Miss M. Baxter, M. Gregson, I. Ingles, L. Kelly, M. Lavie, A. McAndrew, L. May, C. Morrin, T. Patrick, B. Taylor G. Thomas, H. Tompkins, K. Wallbank, E. Williamson, Mrs. Helen Penney, Mrs. Louis Price, Mrs. Bertha Priestley, Q.A.I.M.N.S. (R.); Misses M. Cox, S. Jubb, E. Skerratt, G. Walford, Mrs. Mary Mundy, C.N.S.; Misses M. Johnston, E. Midgley, M. Paul, and E. Smeeton, T.F.N.S.; Misses M. Pidcock-Henzel, J. Millar, E. Pinkerton, G. Sutton, M. Wilson, F. Wren, Mrs. Cora Mayne, Mrs. Una Dawson-Pattisson, Mrs. Alice Scott, B.R.C.S.; Miss M. Peter, St. John Ambulance Brigade; Misses A. Carr, E. Jones, E. Myers, W. Taylor, M. Williams, Mrs. Bertha Parry, Civil and War Hospital; Misses D. Jobson, A. Kearney, L. Roskill, M. Wigram, V. Wigram, Mrs. Zoe Longdon, Mrs. Gertrude Ross, Mrs. Marion Sadler, The Hon. Mrs. Eva John Trefusis. Queen Alexandra received all the above ladies together with Miss A. B. Smith, R.R.C., at Marlborough House after the Investiture.

At the second Investiture held on March 25 the King personally conferred decorations as follows:—Bar to the Royal Red Cross: Miss Amy Knaggs, Q.A.I.M.N.S. Royal Red Cross, 1st Class: Misses C. Harries and K. Bulman, Q.A.I.M.N.S.; Miss K. Chapman, Q.A.I.M.N.S. (R.); Miss E. Humphries, T.F.N.S.; Mrs. Phyllis Dry and Miss L. Holroyde, C.N.S.; Mrs. Josephine Latter and Lady Maude Fox-Symons, V.A.D. Royal Red Cross, 2nd Class: Misses H. King and I. Kinkead, Q.A.I.M.N.S.; Misses A. Clarke, C. Clements, E. Cooke, C. Dimmock, G. Fuller, G. Gilfillan, B. Hacker, F. Hepburn, H. Howard, E. Johnson, A. Langley, I. Leedam, P. Le Gassick, L. Leigh, A. Letts, A. Lewis, B. Lidstone, D. Lynch, and Mrs. Elizabeth Rickard, Q.A.I.M.N.S. (R.); Misses M. Dow-Bain, E. Bradshaw, M. Bulteel, A. Cameron, F. Egginton, M. Harris, M. Haynes, E. Hobday, M. Kitteringham, S. Leavesley, J. Lyle, and Mrs. Mabel Quormby, T.F.N.S.; Misses M. Bean, R. Cooper, E. George, E. Gooderham, and A. Lawler, C.N.S.; Misses M. Holbeach, M. Kirk, I. Morrison, and Mrs. Marguerite Johnston, B.R.C.S.; Misses D. Ashbridge, B. Bayley, H. Brownrigg, C. Douglas, C. Germon, S. Harries, E. Drummond-Hay L. Lamonby, H. Lee, E. Lewis, M. Maberley, I. Megson, J. Laing, M. Sedgwick, Mrs. Mildred Gray, Mrs. Janet Hallsmith, Mrs. Gertrude Ravenscroft, Mrs. Mabel Barr-Stevens, V.A.D. Queen Alexandra received all the above ladies with Dame Sidney Browne at Marlborough House after the Investiture.

The Investitures will be continued after Easter for some considerable length of time. It is stated that there are still 25,000 persons who have not received the Orders and decorations awarded them for war service, and the King is desirous of conferring the honour personally as far as possible.

Nurses will be interested to learn that the Ministry of Labour has asked the College of Nursing to draw up a scheme of hours under which nurses could be included in the Hours of Employment Bill. The Parliamentary Committee has drafted such a scheme, which has been forwarded to the local centres for the members' considered opinion thereon.

The Ministry of Health for a second time has refused to allow the Lambeth Board of Guardians to engage probationer nurses who may reside outside the infirmary. The Ministry, in its last letter, stated that the balance of advantage is definitely against the introduction of such a scheme, and in these circumstances the Minister of Health is not prepared to revise his previous decision. Nothing daunted, the guardians have now decided to appoint a deputation to wait on Dr. Addison to see if they cannot by personal argument induce him to give their scheme a trial before he condemns it root and branch. In reply to their advertisement for probationer nurses to live out the guardians received no fewer than 74 applications for the posts, which show that there are many willing to adopt the nursing profession provided that they are not compelled to reside in the institution. The guardians were forced to advocate the employment of non-resident probationers because of the very poor response they had to their appeal for resident probationers.

The great hold which the *Daily Telegraph* Shilling Fund has upon the nursing world is evidenced by numerous subscriptions sent this week from various hospitals and centres of the College of Nursing. The *Leicester Mail* has generously followed the example set by their London contemporary, and has already forwarded two contributions, each of 2,000 shillings, in response to appeals made in that paper. It is quite evident that immediate success attends the efforts of all who take the trouble to collect on behalf of the Fund. We suggest that nursing sisters should interest their friends and relatives in the movement and induce them to undertake this duty. Nine persons out of ten will do what they are asked to do by one whom they cordially respect, and many women take a real pleasure in collecting; yet very few will undertake it without being personally invited to do so. "Those who organise such collections," says the *Daily Telegraph*, alluding to the sums collected by firms, "will find little difficulty in obtaining subscriptions," and this

**ROUND THE HOSPITALS—(*continued*).**

has been found to be true all along the line. Let every nurse, then, set herself to find a collector and the Fund will grow apace. The amount contributed as we go to press amounts to 221,278 shillings.

The increased cost of living, and the extra demands made by rising salaries on the hospital exchequer, must not make us oblivious of our good fortune in this country as regards prices when compared with other countries. In the recent plea for funds to reopen the Queen Alexandra Sanatorium at Davos Platz, Lord Balfour of Burleigh and his coadjutors called attention to the fact that up to the outbreak of war the sanatorium provided first-rate accommodation and treatment for eighty British patients of small means at the very low fee of £2 2s. weekly. It has been closed during the war and the Council, in their efforts to reopen it, are faced with an enormous increase in the cost of maintenance. Estimates of the cost of each patient weekly under present prices vary between £5 5s. and £7 7s., and "are not very reliable." Let hospital managers consider what plight they would be in if this rate prevailed in Great Britain. The expenses incidental to reopening, repairs, renewals, and stocks of fuel, are estimated at about £7,000, and this comparatively small sum should speedily be forthcoming if for no other reason than "to prevent the loss of prestige which might follow if such an important British institution failed to open its doors again for lack of support from home." Donations or promises of help may be sent to Mr. Edward C. Simmons, 18 Finch Lane, Cornhill, E.C. 3.

It is high time that the Central Midwives Board should bow to the prevailing passion for a distinctive decoration and authorise the issue of a registered midwives' badge. Such a badge would contribute to raise the status of the midwife both in rural and urban localities. It would prove a highly useful adjunct to the uniform of the qualified health visitor and maternity centre worker, and often act as a passport in private and maternity nursing. The midwife frequently has to work with colleagues who do not possess the C.M.B. certificate, and is surely entitled to wear a badge which testifies to her knowledge and dispenses her from the necessity of insisting upon her qualifications. We are quite sure that if the midwifery inspectors in every county bestirred themselves to get petitions signed in favour of this concession the general desire for the badge would speedily be made manifest.

Much criticism, favourable and the reverse, has been poured out on London's new statue of Edith Cavell. So bold a conception and massive design must inevitably produce a powerful effect, and in such cases the first view, disturbing some preconceived notion in the mind of the spectator, is often a shock. More and more, however, does the figure grow on those who pass by. The face, always the test of a statue, is remarkably impressive. It is the crystallisation of that heroic mood in which Edith Cavell lent herself to the rescue of her countrymen, and went undismayed, without rancour, to her death. To make that spirit live in marble for future generations is the supreme achievement of the sculptor.

The War Library, which did so much to cheer the sick and wounded, has reorganised itself under the rather cumbrous title of the British Red Cross and Order of St. John Hospital Library, and is shedding its light on many civilian hospitals, where books are scarce. We venture to plead for gifts of children's books, out-of-date magazines, and shabby volumes of any description which can be baked or burnt without remorse for the little isolation hospitals dotted about the country. Usually there is a long convalescence with nothing whatever to do, for these establishments are bare of every form of recreation and are never visited. Spare books, magazines, and illustrated papers are welcomed for distribution at the offices of the Library, 48 Queen's Gardens, Lancaster Gate, W. 2.

The death is reported of the Hon. Evelina Haverfield, who was one of the brave volunteers who went with the Scottish Women's Hospital to combat typhus in Serbia in 1915. She spent the whole of that time, in its unspeakable wretchedness, with the sick and wounded in Krushevatz, and was taken prisoner by the Germans. After her repatriation she went as commandant of the motor ambulance and transport with Dr. Inglis to South Russia, and took part in the terrible retreat from the Dobrudja. She was the originator of the Haverfield Fund for Disabled Serbian Soldiers, and in 1918 she went once more to Serbia, where she started orphanages for the desolate children whose fathers had been killed. She fell a victim to pneumonia in the midst of her labours of love, and has left a name behind distinguished among her many brave compeers in the Scottish Women's Hospital for courage, wisdom, and self-devotion.

The Council of Queen Victoria's Jubilee Institute for Nurses met at 58 Victoria Street, with Lord Athlone in the chair, and appointments made by Queen Alexandra to the new triennial Council were reported. The Council is a large one, and, in addition to the members appointed by the President, contains many representatives of various associations. The same is the case with the Executive Committee, on which members representing the affiliated associations, the superintendents of the Queen's nurses, and other kindred societies find a place. Princess Christian, Princess Louise (Duchess of Argyll), and Princess Beatrice are among Queen Alexandra's nominees, with seventeen others.

The immense value of the Ladies' Linen League to every hospital where one is in operation has often been demonstrated. The Royal Albert Edward Infirmary, which projected a Linen and Needlework Guild the year war broke out, has at length secured

**ROUND THE HOSPITALS—(*continued*).**

the help of the ladies of Wigan in this direction. The Guild had an enthusiastic beginning at its inaugural meeting, when £75 was subscribed, and Miss M. K. Coggins, the Matron of the Infirmary, has the pleasure of realising a long-delayed scheme. Working members are to pay 5s. a year and undertake to make three garments as required; honorary members pay £1 1s. a year. The Countess of Crawford and Balcarres was appointed President; Lady Gerard, Vice-President; Mrs. E. Lawrence, O.B.E., Chairman; Mrs. Harold Sumner, Hon. Treasurer. The annual exhibition at Brighton of the Blanket, Linen and Work Guild connected with the Sussex County Hospital illustrates the extent and variety of the aid afforded by these means. A great effort was made, at the request of the Matron, Miss K. Scott, to restock the linen store this spring, and the expenditure of the Guild on blankets, linen, etc., exclusive of the articles presented, amounted to £535. The subscriptions, alas! reached the lowest point on record, and are £200 less than in 1913.

---

A fire which might have proved disastrous had it not been for the presence of mind of the nurses broke out early on Friday morning at the College annexe of St. Dunstan's Hostel for Blinded Soldiers and Sailors, Regent's Park. Everyone took things calmly, and all the patients, who numbered 150, were assisted to a place of safety without a moment's confusion or delay. The fire broke out on the first floor, and on the top storey fifty V.A.D. nurses were sleeping. The real danger was here, as the staircase was cut off, but, roused in time, all made their escape by the emergency iron staircase outside, though the fire gained so fast that many had only time to get on their cloaks and shoes. Two were injured in the hurried descent, and one, Nurse Frankland, was removed to the Middlesex Hospital suffering from the effects of the suffocating smoke. The sisters are stated to have marshalled the blind patients with the utmost coolness, and they on their part displayed the alertness of mind and body which is the leading characteristic of St. Dunstan's men.

---

The resident medical officer of Westminster Hospital awoke a responsive feeling in many by his recent complaint of the intolerable anomaly of extending gratuitous hospital treatment to highly-paid manual workers, while the doctors often find it difficult to make both ends meet, and the well-trained ladies of the nursing staff are rewarded with "a salary at which a drunken cook would turn up her nose." The extension of pay-wards is merely a matter of time and reorganisation. It has often been advocated in these columns. It is now being rapidly forced upon hospital managers. But it involves a danger of loss to the nursing profession unless the position of the nursing sisters in the pay-wards can be safeguarded. They ought not to be in a worse position than when nursing private patients in their own houses. If the American system of pay hospitals is to take root in this country it must be seen to that the nursing sisters, whose part it will be to carry out the internal administration, get something better in the way of hours and appointments than would content the cook, even when sober.

---

At the annual meeting of the Colchester and District Nursing Association, with the Mayor presiding, it was reported that the superintendent nurse was granted six months' leave last year to enable her to go to her previous work in Sierra Leone to relieve the workers of the mission in that place. The nurses received high praise from Dr. Corfield as pioneers of health work, reaching people the Health Department could not reach, and preaching wherever they went the gospel of the prevention of disease. The present minimum fee for the nurses' services is 6d. a visit, and we agree with one of the speakers, Mr. Millican, that the fee is far too low if the general public are permitted to enjoy this privilege. The charge of 1s. a night, which is laid down as proper for night nursing by some County Associations, is even less defensible. A great deal of harm is done by charging these low fees, which people are thereby educated to believe are adequate.

---

Ancoats Hospital is among the numerous institutions at the present moment appealing for funds to build a new Nurses' Home. The amount required is no less than £50,000, and the sum promised so far is little more than a tenth. Lord Cawley, the President of the hospital, stated at the annual meeting a short time ago that better accommodation for the staff had become an absolute necessity. The rooms which will be released in the hospital by providing a separate home for the nurses are urgently needed for the ever-increasing medical and surgical requirements.

---

The Hull General Infirmary is among the numerous institutions which have outgrown their Nurses' Homes. At the 137th annual meeting on March 18 Mr. C. D. Holmes, who presided, announced that the Board had decided to increase the accommodation for nurses. Fortunately, the Watts' Home can be added to either by putting on a third storey or in some other way, and thus room will be provided for the additional nurses required to carry out the reduction of working hours lately decided on. The cost of providing more nurses is estimated at £1,000 a year, and this is over and above the increase shown by additions to salaries. Cordial allusions were made by more than one speaker to the hard work and cheerfulness of the nursing staff as a reason for lightening their labours by shorter hours. In particular, the chairman of the Working Men's Committees was able to testify by actual experience to the skill of the nurses, having been himself an in-patient during the past year. It is significant that the working-men's contributions have gone up from £6,000 to £9,000, or 50 per cent., a sure test of the popularity of the institution.

